# A Toxin-Antitoxin System VapBC15 from *Synechocystis* sp. PCC 6803 Shows Distinct Regulatory Features

**DOI:** 10.3390/genes9040173

**Published:** 2018-03-21

**Authors:** Qian Fei, E-Bin Gao, Biao Liu, Yao Wei, Degang Ning

**Affiliations:** 1 CAS Key Laboratory of Algal Biology, Institute of Hydrobiology, Chinese Academy of Sciences, Wuhan 430072, China; 18362545515@163.com; 2 College of Life Sciences, University of Chinese Academy of Sciences, Beijing 100049, China; 3 School of the Environment and Safety Engineering, Jiangsu University, Zhenjiang 212013, China; gaofei@ujs.edu.cn (E-B.G.); liu502306@126.com (B.L.); 4 Huai’an Research Center, Institute of Hydrobiology, Chinese Academy of Sciences, Huai’an 223005, China; 18352862463@163.com

**Keywords:** cyanobacteria, *Synechocystis* PCC6803, type II toxin-antitoxin system, VapBC15, regulatory feature

## Abstract

Type II toxin–antitoxin (TA) systems play important roles in bacterial stress survival by regulating cell growth or death. They are highly abundant in cyanobacteria yet remain poorly characterized. Here, we report the identification and regulation of a putative type II TA system from *Synechocystis* PCC6803, VapBC15. The VapBC15 system is encoded by the chromosomal operon *vapBC15*. Exogenous expression of VapC15 dramatically arrested cell growth of *Escherichia coli* and reduced the numbers of colony-forming units (CFU). The VapC15 toxicity could be neutralized by simultaneous or delayed production of VapB15. Biochemical analysis demonstrated the formation of VapB15-VapC15 complexes by the physical interaction between VapB15 and VapC15. Notably, the VapB15 antitoxin up-regulated the transcription of the *vapBC15* operon by directly binding to the promoter region, and the VapC15 toxin abolished the up-regulatory effect by destabilizing the binding. Moreover, VapB15 can be degraded by the proteases Lons and ClpXP2s from *Synechocystis* PCC6803, thus activating the latent toxicity of VapBC15. These findings suggest that VapBC15 represents a genuine TA system that utilizes a distinct mechanism to regulate toxin activity.

## 1. Introduction

Toxin-antitoxin (TA) systems consist of a stable toxic protein and its labile cognate antitoxin, which are generally encoded by a dicistronic operon. Originally, TA genes were identified on plasmids as addiction modules to ensure stable plasmid maintenance in bacterial populations [1]. With the increase in the number of sequenced genomes, TA modules have been found on almost all prokaryotic chromosomes [2,3,4]. The prevalence of chromosomal TA genes suggests their important physiological functions since bacterial chromosomes have no need for an addiction module [5,6].

To date, six types of TA systems have been identified based on the nature of the antitoxin and its mode of action. Type I and type III are characterized by a small non-coding RNA as the antitoxin [7,8,9,10], and types II, IV, V, and VI feature proteinaceous antitoxins [11,12,13]. Among these, type II TA systems are the most numerous and best characterized. The toxins of TA systems can slow down or inhibit cell growth or even kill a cell via targeting a variety of vital cellular structures and functions, such as membrane integrity, replication, cell wall synthesis, ribosome assembly, and translation factors [11,14,15]. Based on the biochemical activities of toxins, type II TA systems are further classified into several families [16]. For example, VapC-family toxins harbour a conserved PilT N-terminus (PIN) domain and function as substrate- and sequence-specific endoribonucleases [17,18,19]. The proteinaceous antitoxins of type II TA systems are unstable because of their susceptibility to adenosine triphosphate (ATP)-dependent proteases [11,14]. The antitoxins generally contain two domains: a toxin-binding domain with an unstructured fold and a DNA-binding domain belonging to Helix–Turn–Helix (HTH), Ribbon–Helix–Helix (RHH), AbrB, or Phd/YefM class [14]. The toxin-binding domain in antitoxins is involved in neutralizing the toxic function of their cognate toxins by forming TA complexes, and the DNA-binding domain mediates the transcriptional auto-regulation of their own operons. Usually, type II antitoxins auto-repress their own promoter both alone and, even more effectively, in complexes with their cognate toxins [11,14]. Nevertheless, the transcriptional regulation of some TA systems is dictated by the stoichiometry between toxin and antitoxin proteins, termed conditional cooperativity [20,21,22].

Therefore, expression and activation of TA systems are tightly regulated by their cognate antitoxins at both transcriptional and post-transcriptional level [6,15,23]. Under favorable growth conditions, the co-expression of an antitoxin in excess of its cognate toxin causes auto-repression of TA expression and formation of non-toxic TA complexes, thus preventing the toxin from slowing down or inhibiting cell growth. However, when environmental stresses and physiological changes occur, intracellular physiologic changes such as the rates-of-translation reduction, or even protease activation would cause decreases in cellular levels of antitoxins due to proteolytic degradation. As a result, the auto-repression of TA expression is relieved, and the more stable toxin is released from TA complexes [8]. The activated toxin regulates cell growth, allowing cells to enter a stress-tolerant state until more favorable environmental conditions return [14,19,24]. Recent investigations have suggested that the regulation of toxin activity may be more subtle and complex than initially expected [18,25].

Development of computational approaches have facilitated identification of type II TA systems [3,4,16]. For example, a bloom-forming cyanobacterium *Microcystis aeruginosa NIES-84* was predicted to have the largest number (as many as 113) of type II TA systems [3]. A model cyanobacterium, *Synechocystis* PCC6803 (hereafter, *Synechocystis*), also was believed to have 69 TA operons on the chromosome [26], but only a few were characterized [27,28,29]. Among those, a gene pair *ssr2201* and *slr1327* was predicted to encode a VapBC-family TA system, designated as VapBC15, due to the presence of a PIN domain in the *slr1327*-encoding protein [3,4,16]. A survey of genetic context showed that the *ssr2201* gene (hereafter, *vapB15*) lies immediately upstream of *slr1327* (hereafter, *vapC15*), and overlaps *vapC15* by 11 nucleotides (Figure S1). Recently, both genes *vapB15* and *vapC1*5 were demonstrated to form a dicistronic operon (hereafter *vapBC15*) [30]. However, in the putative TA system, the predicted antitoxin VapB15 does not contain any conserved domain related to the known antitoxins, and its function remains elusive.

A detailed understanding of TA systems is essential for the interpretation of their functions and the exploration of their practical exploitation [31,32]. The aim of this study was to identify the putative TA system VapBC15 of *Synechocysitis* and assess the regulation of toxin activity. Our data show that the *vapBC15* operon encodes a TA system VapBC15 with unusual features. Notably, the VapB15 antitoxin was able to transcriptionally up-regulate its own operon by directly binding to the promoter region, and the VapC15 toxin neutralized the up-regulatory effect by destabilizing the binding. We also show that VapB15 is degraded by both Lons and ClpXP2s proteases, thus causing toxin activation of the VapBC15 system.

## 2. Materials and Methods

### 2.1. Strains, Culture Conditions, and Biochemicals

All strains and plasmids used in this study are listed in Table 1. *E. coli* strains were grown in the liquid Luria Bertani Broth (LB) medium or on agar plate unless otherwise noted. When required, media were supplemented with ampicilin (Ap, 50 mg/L), spectinomycin (Sp, 100 mg/L), or kanamycin (Km, 50 mg/L).

All enzymes were purchased from Takara Biotech (Dalian, China). [γ-^32^P] ATP was obtained from Furida Biotech (Beijing, China). Nickel-Nitriloacetic Acid (Ni-NTA) resin was purchased from Invitrogen (Carlsbad, CA, USA). Horseradish peroxidase-labeled goat anti-rabbit immunoglobulin G (IgG) conjugate and chemiluminescence reagent were obtained from Beyotime Biotech (Haimen, China). Polymerase chain reaction (PCR) primers were synthesized by Sangon Biotech (Shanghai, China) and listed in Table S1.

### 2.2. Plasmid Construction

The plasmid pJS298 [27], containing a isopropy-β-d-1-thiogalactoside (IPTG)-inducible promoter *P_T7lac_* and a arabinose-inducible promoter *P_BAD_*, was employed for selective expression of VapB15 and/or VapC15. The *vapC15* gene was amplified by PCR from *Synechocystis* chromosomal DNA using the primer pair slr1327-N and slr1327-K. The product was digested with *Nde*I and *Kpn*I, and then cloned behind the promoter *P_T__7lac_* in pJS298 generating pJS307. The *vapB15* gene was amplified using the primers ssr2201-S and ssr2201-K. The resulting fragment was digested with *Sac*I and *Kpn*I and placed under the promoter *P_BAD_* in pJS307-generating pJS357.

For the co-expression of VapB15 and VapC15, the fragment containing *vapB15* and *vapC15* was PCR-amplified with the primers ssr2201-N and slr1327-X. After digestion with *Nde*I and *Xho*I, the resulting fragment was cloned into pET30a generating pJS666, which allows the co-expression of VapB15 and the C-terminally hexa-histidine-tagged VapC15 (VapC15-His_6_) in the IPTG-induced cells of *E. coli* BL21(DE3).

To construct transcription fusions of the *vapBC15* elements and β-galactosidase gene (*lacZ*), the reporter plasmid pJS759 [28] was used. The promoter of the *vapBC15* operon (*P_vapBC15_*) was computationally analyzed using the TSSP software (SoftBerry, Mt. Kisco, NY, USA, http://www.softberry.com), and the promoter fragment was amplified using the primers ssr2201-1 and ssr2201-2, and cloned into pMD18-T (Takara Biotech) in the opposite direction of *lacZ* generating pJS694. The cloned fragment was excised from pJS694 with *Hin*dIII and *Eco*RI, blunted with T4 DNA polymerase, and then linked to pJS759 which was digested with *Bgl*II and blunted similarly, generating pJS778 (containing the transcription fusion *P_vapBC15_-lacZ*). The fragments containing *P_vapBC15_-vapB15* and *P_vapBC15_*-*vapB15-vapC15* were amplified with the primer pairs ssr2201-1/ssr2201-K and ssr2201-1/slr1327-X, respectively. The amplified fragments were cloned into pMD18-T in the opposite direction of *lacZ*, generating pJS766 and pJS956. The fragments *P_vapBC15_-vapB15* and *P_vapBC15_*-*vapB15-vapC15* were released from pJS766 and pJS956 with *Pst*I and *Kpn*I, and linked to pJS778 digested with the same restriction enzymes, producing pJS779 (containing the fusion *P_vapBC15_-vapB15-lacZ*) and pJS962 (containing the fusion *P_vapBC15_-vapB15-vapC15-lacZ*), respectively.

To construct the plasmids for selective expressing VapBC15 components and *Synechocystis* proteases, the vectors pJS371 and pJS391 [27] were employed. The fragment containing the genes *vapB15* and *vapC15* was amplified using the primers ssr2201-S and slr1327-K, digested with *Sac*I and *Kpn*I, and then cloned behind *P_BAD_* in pJS371 and pJS391 generating pJS744 and pJS745, respectively. The *vapB15* gene was amplified using the primers ssr2201-S and ssr2201-K, digested with *Sac*I and *Kpn*I, and sub-cloned under *P_BAD_* in pJS371 and pJS391, producing pJS913 and pJS914, respectively.

### 2.3. Analyses of Toxicity, Anti-Toxicity, and Growth Rescue

To investigate toxicity, anti-toxicity, and proteolytic activation of VapC15, drop dilution tests were conducted using *E. coli* BL21(DE3) harboring the corresponding plasmids. Cultures were grown to an optical density at 600 nm (OD_600_) of about 0.6 in liquid LB medium supplemented with 0.2% glucose. Subsequently, cells were collected by centrifugation, washed twice with liquid M9 medium with 0.2% glycerol. The washed cells were resuspended to an OD_600_ of 0.2 in liquid M9 medium with glycerol and subjected to 10-fold serial dilution. A 5 μL portion of each diluted sample was dropped on the M9 agar plates containing 0.2% (w/v) glycerol (indicated as M9 + Gly), supplemented with 0.1 mM IPTG (indicated as M9 + Gly + IPTG), 0.2% (w/v) arabinose (indicated as M9 + Gly + Ara) or both (indicated as M9 + Gly + IPTG + Ara) for selective expression of genes under control of the promoters *P_T7__lac_* and/or *P_BAD_*. The plates were incubated at 37 °C for 20 h.

For growth rescue experiments, cultures were grown to an OD_600_ of about 0.6 in liquid LB medium containing 0.2% glucose and then supplemented with 0.1 mM IPTG. At time zero and subsequent time points, aliquots were taken, spun down, and washed twice with liquid M9 medium with glycerol. The washed cells were resuspended in appropriate volume of liquid M9 medium with glycerol, and the final concentrations were adjusted to an OD_600_ of 0.6. A 100 μL portion of each diluted sample was were spread on the agar plates M9 + Gly, M9 + Gly + IPTG and M9 + Gly + Ara, respectively. After incubation at 37 °C for 30 h, the colony-forming units (CFUs) were counted.

### 2.4. Co-Expression, Purification and Antibody Preparation of VapB15 and VapC15

The logarithmic-phase culture (OD_600_ of about 0.6) of *E. coli* BL21(DE3) containing the co-expression plasmid was induced with 1 mM IPTG for about 3 h. The cells were collected and sonicated in ice-cold lysis buffer (50 mM NaH_2_PO_4_, 0.3 M NaCl, 10 mM imidazole, 5 mM β-mercaptoethanol, pH 8.0). To co-purify the VapB15-VapC15-His_6_ complex, the cleared lysate was subjected to affinity chromatography using Ni-NTA resin under native conditions according to the manufacturer’s instructions. The co-expressed proteins VapB15 and VapC15-His_6_ were individually purified and renatured as previously described [33]. Briefly, the Ni-NTA agarose bound with the VapB15-VapC15-His_6_ complexes was sequentially eluted using alkaline denaturing buffer (100 mM NaH_2_PO_4_, 10 mM Tris-HCl, 8 M Urea, pH 8.0) and acidic denaturing elution buffer (100 mM NaH_2_PO_4_, 10 mM Tris-HCl, 8 M Urea, pH 4.5). The eluted proteins were refolded by sequential dialyses: once with PBS (pH 8.0), 0.1% (*v*/*v*) Triton X-100, 5 mM DTT; once with PBS (pH 8.0), 5 mM DTT; once with PBS (pH 8.0), 20% (*v*/*v*) glycerol, 5 mM DTT. The concentrations of proteins were determined by the Bradford method.After the purified proteins were separated on 18% Sodium dodecyl sulfate-polyacrylamide gel electrophoresis (SDS-PAGE), the bands of co-purified proteins were excised and subjected to matrix-assisted laser desorption/ionization-time-of-flight (MALDI-TOF) mass spectrometry analysis as previously described [34]. The predicted peptide fingerprint mass mappings of the co-purified proteins were obtained using ProteinProspector Tools (http://prospector.ucsf.edu/prospector/mshome.htm). Polyclonal antibodies were produced by immunizing New Zealand rabbit with the purified proteins [27].

### 2.5. Assay of β-Galactosidase (LacZ) Activity

Assay of β-galactosidase activity was performed in *E. coli* DH5α cells containing the corresponding transcription fusion plasmids. The cells were cultured at 37 °C to an OD_600_ of about 0.5, and the β-galactosidase activity was measured and calculated as previously described [28].

### 2.6. Electrophoretic Mobility Shift Assay (EMSA)

The DNA fragments used for analysis of DNA-binding activity were prepared using PCR amplification with the respective primers. The resulting fragments were purified and labeled with T4 polynucleotide kinase and [γ-^32^P] ATP following the manufacturer’s instructions. The mixture was treated at 65 °C for 7 min to inactivate the kinase in the reactions. For specific and nonspecific binding tests, 1 μM of the unlabeled fragment was used as competitor DNA. The labeled DNA fragments were incubated at 25 °C for 30 min with different amounts of proteins in a total volume of 20 μL EMSA buffer (100 mM Tris-HCl, pH 8.0, 100 mM NaCl, 1 mM DTT and 10% glycerol) with 0.1 mg/mL sonicated salmon sperm DNA. The mixtures were then subjected to 5% native PAGE containing 0.5 × Tris–borate–EDTA buffer at 150 V at room temperature for about 1.5 h. Images were acquired by a storage phosphor screen (GE Healthcare, Little Chalfont, UK).

### 2.7. Western Blot Analysis

The *E. coli* cells harboring corresponding expression plasmids were cultivated to an OD_600_ of about 0.5 in the presence of 0.2% arabinose, and then supplemented with 0.1 mM IPTG. After induction for 30 min (at time zero), protein synthesis was inhibited by adding 100 μg/mL spectinomycin. Aliquots were taken from the induced cultures at a 10-min interval for 50 min. An equal amount of cells (approximately 10^8^) was individually collected from the samples, and lysed by boiling in 50 μL of lysis buffer (20 mM Tris–HCl, 150 mM NaCl, pH 8.0) and 50 μL of 2 × SDS-gel loading buffer (Takara) for 10 min. Ten micrograms of each protein sample were separated on 15% SDS-PAGE gels and then transferred to a poly-vinylidene fluoride (PVDF) membrane. The membrane was incubated with a 1:1000 dilution of antibodies at room temperature for 1 h, followed by 1 h incubation with a 1:2000 dilution of horseradish peroxidase–labeled goat anti-rabbit IgG. The immunoreactive bands were visualized with enhanced chemiluminescence reagents and exposed to an X-ray film. Images from exposed films were analyzed densitometrically using ImageJ [35].

## 3. Results

### 3.1. Growth-Arrest Effect of VapC15 and Toxicity-Neutralization Effect of VapB15

**Figure 1:**
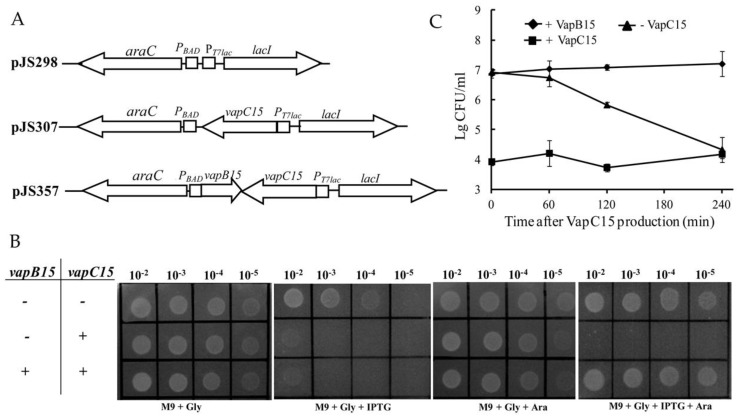
Effects of VapB15 and VapC15 on *E. coli* growth. (**A**) Schematic diagram illustrating the structure of the selection expression plasmids. (**B**) Drop dilution assays for the effects of ectopic production of VapBC15 components on *E. coli* growth. + or − indicates the presence or absence of the *vapBC15* genes in *E. coli* BL21(DE3) cells, (**C**) Growth-rescue analyses for toxic effect of VapC15 using *E. coli* BL21(DE3) cells containing pJS357. + indicates the induction of VapB15 or VapC15 expression. − indicates the stop of VapC15 expression. Data points represent the means of three independent cultures and the error bars represent standard deviation (SD).

Effects of VapB15 and VapC15 on cell growth were investigated by drop dilution assay using *E. coli* BL21(DE3) harboring the corresponding expression plasmids (Figure 1A). The plasmid pJS307 contains the gene *vapC15* alone under control of the promoter *P_T7lac_*, while pJS357 contains both genes *vapC15* and *vapB15* under control of the promoters *P_T7lac_* and *P_BAD_*, respectively. As seen in Figure 1B, the *E. coli* cells in the absence of *vapBC15* genes (pJS298) grew well on all test agar plates. However, the presence of *vapC15* alone (pJS307) allowed *E. coli* cells to grow on the agar plates M9 + Gly and M9 + Gly + Ara but did not on M9 + Gly + IPTG and M9 + Gly + IPTG + Ara. In the presence of *vapC15* with *vapB15* (pJS357), the *E. coli* cells could grow on M9 + Gly, M9 + Gly + Ara and M9 + Gly + IPTG + Ara but could not on M9 + Gly + IPTG. Also, similar growth profiles of these strains were observed in liquid media with the corresponding inducers (data not shown). These results indicated that the production of VapC15 inhibited cell growth and VapB15 blocked the growth-arrest effect of VapC15. Thus, VapB15 and VapC15 constitute a type II TA system where VapC15 is the toxin and VapB15 is the antitoxin.

### 3.2. VapB15 Rescues the Growth Arrest Induced by VapC15

Previous studies showed that the toxic effect of some toxins is bacteriostatic, while others’ is bactericidal [27,36,37,38,39]. To further determine the toxic effect of VapC15, growth-arrest rescue experiments were conducted by stopping VapC15 production or by inducing VapB15 expression using the *E. coli* BL21(DE3) strain with pJS357. For this, the viability of IPTG-induced cells was assessed via quantifying CFU on the agar plates M9 + Gly, M9 + Gly + IPTG, and M9 + Gly + Ara. As shown in Figure 1C, the stop of VapC15 production (on the plate M9 + Gly) did not cause a reduction in CFU at 60 min post pre-induction of VapC15 expression; however, approximate 10- and 1000-fold reductions were observed at 120 and 240 min after VapC15 production, respectively. The constant production of VapC15 (on the plate M9 + Gly + IPTG) caused an approximately 1000-fold drop in CFU during the whole test period. However, no CFU reduction was observed for the cells that had expressed VapC15 and subsequently expressed VapB15 (on the plate M9 + Gly + Ara). Therefore, the VapC15-induced growth arrest could be rescued by stopping VapC15 production only within a narrow window of time (0–60 min after induction) or by subsequently expressing VapB15 during the whole experimental period. These results suggest that the growth-inhibitory effect of VapC15 is bacteriostatic rather than bactericidal.

### 3.3. Physical Interaction between VapB15 and VapC15

**Figure 2:**
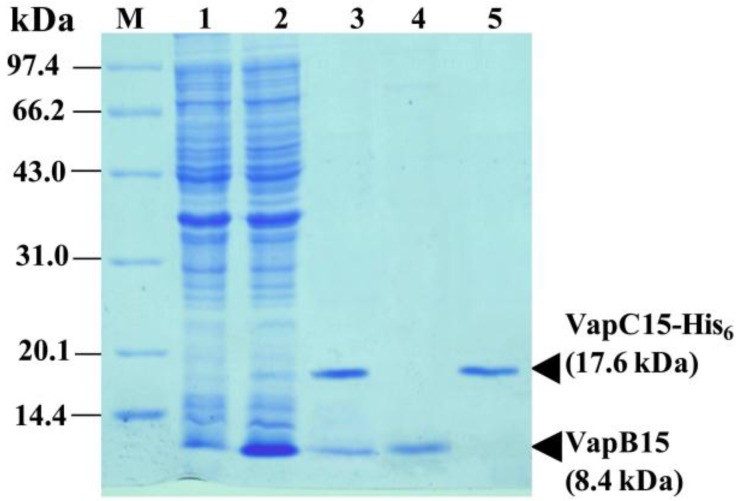
Sodium dodecyl sulfate-polyacrylamide gel electrophoresis (SDS-PAGE) analysis for the recombinant proteins from the isopropy-β-d-thiogalactoside (IPTG)-induced cells of *E. coli* BL21(DE3)(pJS666). **M**, protein molecular weight standard; **1**, uninduced cells; **2**, IPTG-induced cells; **3**, Co-purified proteins by Nickel-Nitriloacetic Acid (Ni-NTA) affinity chromatograph under native conditions; **4** and **5**, Purified and renatured proteins VapB15 and VapC15-His_6_, respectively.

Type II antitoxins counteract their cognate toxins by forming non-toxic TA complexes via the physical interaction between them [14]. We determined the interaction between VapB15 and VapC15 by affinity chromatography combined with mass spectrometry analysis using IPTG-induced *E. coli* BL21(DE3)(pJS666) cells and Ni-NTA resin. As shown in Figure 2, a 8.4-kDa protein and a 17.6-kDa protein, corresponding to the molecular masses of VapB15 and VapC15-His_6_ respectively, were co-expressed in *E. coli* BL21(DE3)(pJS666) cells (Figure 2, lane 2). The 8.4-kDa protein was successfully co-purified with the 17.6-kDa protein by affinity chromatography under native conditions (Figure 2, lane 3), but only the 8.4- or 17.6-kDa protein was purified under denaturing conditions (Figure 2, lanes 4 and 5). The results of MALDI-TOF mass spectrometry analysis showed that the co-purified 8.4- and 17.6-kDa proteins were VapB15 and VapC15-His6, respectively (Figure S2). Thus, VapB15 and VapC15 form the VapB15-VapC15 complex in vivo by their physical interaction, which may contribute to the prevention of VapC15 toxicity by VapB15 (Figure 1).

### 3.4. Up-Regulation Effect of VapB15 and Neutralization Effect of VapC15

**Figure 3:**
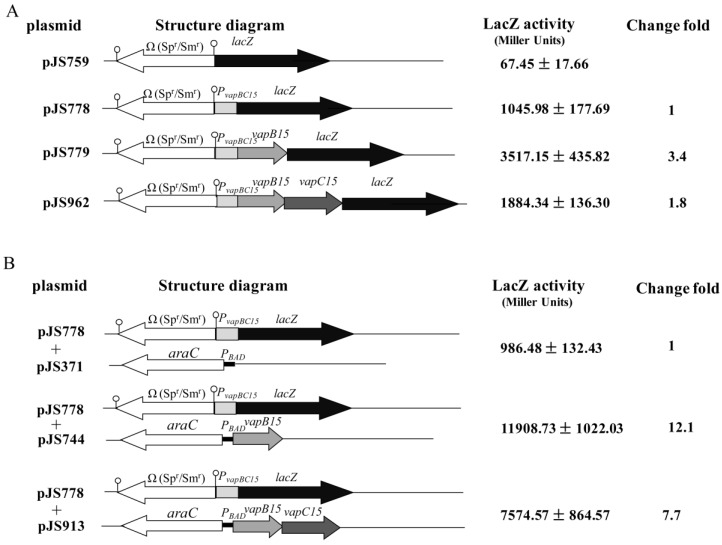
Assays for the auto-regulation of the *vapBC15* operon using the *lacZ* transcriptional fusions. The *E. coli* DH5α cells containing the corresponding plasmids and their LacZ activities are shown. The stemloop symbols at both ends of the Ω cassette indicate the short, inverted repeats that terminate background transcription [40]. (**A**) Regulation in *cis* by *vapB15* and *vapC15*. (**B**) Regulation in *trans*. The change fold is calculated relative to the activities of pJS778 and pJS778 plus pJS371 for regulation in *cis* (**A**) and in *trans* (**B**), respectively. The β-galactosidase activities are presented as means ± SD of three or more independent cultures.

To investigate the regulatory roles of VapBC15 components in the transcriptional activity of their own promoter *P_vapBC15_*, we analyzed LacZ activities from the corresponding *lacZ* transcription fusion plasmids (Figure 3A) in *E. coli* DH5α cells. The results showed that the LacZ activity from the empty vector pJS756 was extremely weak (67.45 Miller units). In the absence of *vapB15* and *vapC15* (pJS778), the basal activity of the *P_vapBC15_* promoter was 1045.98 Miller units. In the presence in *cis* of *vapB15* alone (pJS779), the promoter activity increased approximately 3.5-fold relative to its basal activity. This result indicated that VapB15 up-regulated the transcription activity of the *P_vapBC15_* promoter. However, when both genes *vapB15* and *vapC15* were present in the *lacZ* fusion plasmid pJS962, the LacZ activity increased only 1.8-fold, significant lower than that in the presence of *vapB15* alone (pJS779). This difference between the plasmids pJS779 and pJS962 may be caused by VapC15 that inhibited the auto-regulatory activity of VapB15 or by the different characteristics of the fusion messenger RNAs (mRNAs), such as secondary structure, stability or availability to ribosomes.

To avoid a possible influence of the fusion mRNAs on LacZ production from pJS779 and pJS962, in *trans* donation experiments were conducted. The expression plasmids (pJS371, pJS744 and pJS913, Figure 3B) were introduced individually into *E. coli* DH5α with the *lacZ* fusion plasmid pJS778 (Figure 3A). The co-transformants were cultured in the presence of 0.2% arabinose followed by detection of the β-galactosidase activity from pJS778. The co-transformant containing pJS371 (empty vector) and pJS778 was used to detect the basal activity of *P_vapBC15_* in the absence of VapB15 and VapC15 (pJS778 + pJS371). As shown in Figure 3B, the basal activity of *P_vapBC15_* was 986.48 Miller units, similar to that in the in *cis* donation experiments (pJS778 in Figure 3A). The presence in *trans* of VapB15 alone (pJS778 + pJS913) led to a 12.1-fold increase in the transcription activity of *P_vapBC15_*, while VapB15 plus VapC15 (pJS778 + pJS744) increased only 7.7-fold (Figure 3B). These data further confirmed that VapB15 up-regulated the transcription activity of *P_vapBC15_*, which was partially inhibited by VapC15. Additionally, the in *trans* donation of VapB15 alone or VapB15 with VapC15 caused a higher increase in LacZ activity than the in *cis* donation of those (3517.15 vs. 11,908.73, 1884.34 vs. 7574.57). The differences might result from the influence of the fusion mRNA structure on LacZ production in the in *cis* donate tests.

### 3.5. VapB15 Specifically Binds to the *P_vapBC15_* Promoter

**Figure 4:**
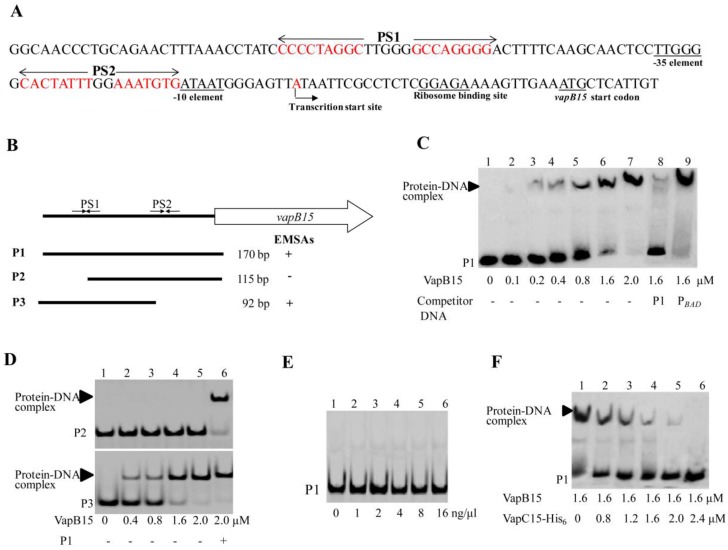
Electrophoretic mobility shift assays (EMSAs) for the binding of VapBC15 components to the *P_vapBC15_* promoter. (**A**) Sequence and elements of the *P_vapBC15_* promoter. (**B**) Schematic representation of the DNA fragments used in EMSAs. The bold lines show the relative position of the DNA fragments P1, P2 and P3. P1, P2, and P3 were prepared by polymerase chain reaction (PCR) using the primers P1-F/ssr2201-2, P2-F/ssr2201-2, and P1-F/P3-R, respectively. + or − indicates whether VapB15 binds to the DNA fragment or not. (**C**) EMSAs for the binding of VapB15 to the *P_vapBC15_* DNA fragment. The labeled fragment P1 was incubated with the final concentrations of VapB15 as indicated below the autoradiograph. The free DNA substrate and DNA-protein complex are indicated on the left. The *P_BAD_* DNA fragment (lane 9) was obtained from pJS298 by PCR amplification using the primers P_BAD_-F and P_BAD_-R and used for nonspecific binding test. (**D**) EMSAs for the sequence required for the binding of VapB15 to the *P_vapBC15_* DNA fragment. The labeled fragments P2 and P3 were incubated with increasing concentrations of VapB15. + or − indicates the presence or absence of the unlabeled fragment P1. (**E**) EMSAs for the binding of the VapB15-VapC15 complex to the *P_vapBC15_* DNA fragment. The labeled fragment P1 was incubated with increasing concentrations of the VapB15-VapC15 complex. (**F**) EMSAs for the effect of VapC15 on the binding of VapB15 to the *P_vapBC15_* DNA fragment. The labeled fragment P1 was incubated with VapB15 pre-incubated with VapC15.

Previous studies demonstrated that TA components auto-repress their own promoters by antitoxin binding to one or more palindrome sequences in the promoter region [25]. To obtain further insights into the *P_vapBC15_* promoter, the promoter elements were analyzed with the RNA structure 4.0 Software and the software TSSP (SoftBerry, Mt. Kisco, NY, USA, http://www.softberry.com). As seen in Figure 4A, both conserved sequences TTGGG and ATAAT are deduced for the -35 and -10 elements with a 17-bp space. In addition, two palindrome sequences (PS), 5′-CCCCCTAGGC-5N-GCCAGGGG-3′ (designated as PS1) and CACTATTT-2N-AAATGTG (designated as PS2) were found at 80 bp and 40 bp upstream of the start code ATG of *vapB15*, respectively. PS1 is located at 17 bp upstream of the -35 element, and PS2 lies just the upstream of the -10 element. Recent RNA sequencing (RNA-Seq) transcriptomic analyses revealed that *vapB15* and *vapC15* was co-transcribed, and the transcriptional start site locates at 28 bp upstream of the start code of *vapB15* [30]. Therefore, these results further confirmed that *vapB15* and *vapC15*, similar as the described TA genes, comprise a transcriptional unit under control of the *P_vapBC15_* promoter.

We first detected whether VapB15 could bind to the *P_vapBC1__5_* promoter by electrophoretic mobility shift assays (EMSAs) using the purified VapB15 (Figure 2, lane 4) as well as the promoter DNA fragment P1 (Figure 4B). The fragment P1 contains both PS1 and PS2. As shown in Figure 4C, when the labeled P1 DNA was co-incubated with increasing concentrations of VapB15 (0, 0.1, 0.2, 0.4, 0.8, 1.6, and 2.0 µM), clear signals of protein-DNA complexes were detected (Figure 4C, lanes 2–7). Further competition assays confirmed the specific binding of VapB15 to its promoter DNA. Unlabeled P1 fragment DNA or unspecific *P_BAD_* promoter DNA were used to compete with the labeled P1. The unlabeled P1 fragment DNA, but not the *P_BAD_* promoter DNA, competitively inhibited the binding of VapB15 to the labeled P1 fragment. These findings strongly suggest that VapB15 can specifically bind to its own promoter region.

Additional EMSAs were performed to identify the sequence required for the binding of VapB15 to *P_vapBC15_* using the labeled fragments P2 and P3. As shown in Figure 4B, P2 contains the 3′ half of PS1 and the whole PS2, and P3 contains the whole PS1 and the 5′ half of PS2. VapB15 was shown to bind to the labeled fragment P3 (Figure 4D, the lower panel) rather than the labeled fragment P2 (Figure 4D, the upper panel). Therefore, the palindromic sequence PS1, but not PS2, is required for VapB15 binding to the *P_vapBC__15_* promoter.

### 3.6. VapC15 Destabilizes the Binding of VapB15 to the *P_vapBC15_* Promoter

To determine whether the VapB15-VapC15 complex can bind to the *P_vapBC15_* promoter, EMSAs were conducted using the purified VapB15-VapC15 complex (Figure 2, lane 3) and the labeled fragment P1 (Figure 4B). As shown in Figure 4E, no signal of protein-DNA complexes was observed, even in the presence of increasing amounts of the VapB15-VapC15 complexes. These results indicated that the VapB15-VapC15 complex could not bind to the *P_vapBC15_* promoter. To further demonstrate the influence of VapC15 on the binding of VapB15 to the *P_vapBC15_* promoter, we conducted EMSAs using the fragment P1 and a constant amount of VapB15 pre-incubated with increasing amounts of VapC15-His_6_. As shown in Figure 4F, the signal of the protein-DNA complex was observed, but gradually weakened with increasing amounts of the pre-incubated VapC15. Therefore, VapC15 was demonstrated to destabilize the binding of VapB15 to the *P_vapBC15_* promoter.

### 3.7. VapB15 Is Susceptble to the Proteases ClpXP2s and Lons

**Figure 5:**
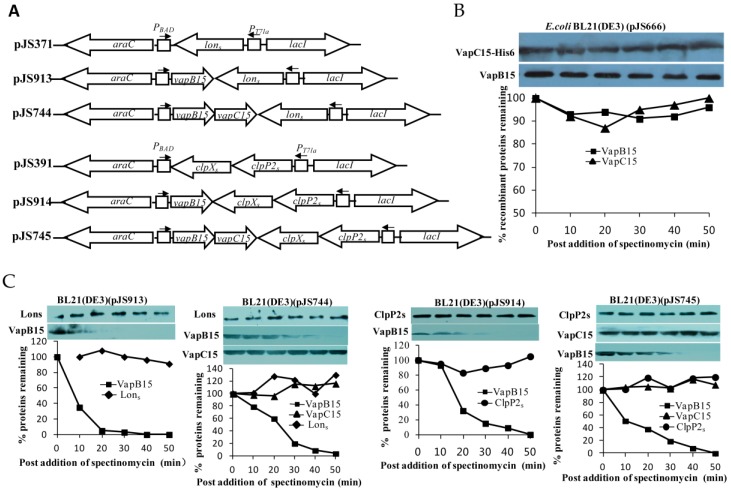
Western blot analyses for the sensitivity of VapB15 and VapC15 to ClpPXP2s and Lons. (**A**) Schematic diagram showing the structure of proteolytic activation plasmids. (**B**) Stability of VapBC15 proteins in *E. coli* cells. The cells of *E. coli* BL21 (DE3)(pJS666) (see Figure 2) were grown, induced and translationally stalled as described in Materials and Methods. The treated cells were subjected to Western blot analysis to monitor VapB15 and VapC15 with the respective antibodies. The graph below represents the percentages of the indicated protein amount at each time point compared to that at time zero. (**C**) Stability of the VapBC15 proteins towards ClpPXP2s and Lons. The *E. coli* cells containing the corresponding plasmids were treated similarly as in (**B**), and subjected to Western blot analysis using the respective antibodies.

The activation of type II TA systems relies on the proteolysis of antitoxins by specific ATP-dependent proteases belonging to the Lon and ClpP families. To test the cleavage of VapB15 by the *Synechocystis* proteases ClpXP2s and Lons, Western blot analyses were conducted using the *E. coli* BL21(DE3) cells containing the corresponding expression plasmids (Figure 5A). We first determined whether or not the *E. coli* proteases could degrade VapB15 and VapC15 using the IPTG-induced *E. coli* BL21(DE3)(pJS666) cells (Figure 2) during spectinomycin-elicited translation inhibition. The results showed that the levels of VapB15 and VapC15 remained relatively stable during translation inhibition (Figure 5B), indicating that the *E. coli* proteases did not degrade VapB15 and VapC15. However, in the cells of *E. coli* BL21(DE3)(pJS913) (expressing Lons and VapB15), the levels of Lons remained stable during translation inhibition, while VapB15 rapidly decreased (Figure 5C). In the *E. coli* BL21(DE3)(pJS744) cells (expressing Lons, VapB15 and VapC15), the levels of Lons and VapC15 remained unchanged during the period of translation arrest, but VapB15 showed a remarkable decrease (Figure 5C). Similar results were obtained in the cells of BL21(DE3)(pJS914) (expressing ClpXP2s and VapB15) and BL21(DE3)(pJS745) (expressing ClpXP2s, VapB15 and VapC15) (Figure 5C). These results suggest that either Lons or ClpXP2s is able to degrade both the free and VapC15-bound VapB15.

### 3.8. Proteolysis of VapB15 Activates the Potential Toxicity of VapBC15

**Figure 6:**
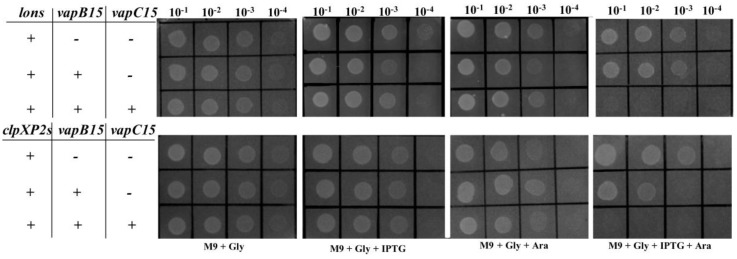
Drop dilution assays for the activation of VapC15 via the proteolysis of VapB10 by Lons and ClpXP2s. + or − indicates the presence or absence of the genes *lons*, *clpXP2s*, *vapB15*, and *vapC15* in *E. coli* BL(21DE3) cells.

Previous studies used ectopic expression of proteases as way to probe the activation of TA systems in *E. coli* [36]. To determine whether or not the proteolysis of VapB15 by Lons or ClpXP2s (Figure 5B) would activate the potential toxicity of VapBC15, drop dilution experiments were conducted using the *E. coli* BL21(DE3) cells harboring the corresponding expression plasmids (Figure 5A). As shown in Figure 6, in the presence of *lons* (pJS371) or *clpXP2s* (pJS745) alone, the *E. coli* cells could grow on all test agar plates. In addition, similar growth profiles were observed in the presence of *lons* (pJS744) or *clpXP2s* (pJS745) with *vapB15*. However, in the presence of *lons* or *clpXP2s* with both *vapB15* and *vapC15*, the cells could grow on the agar plate M9 + Gly, M9 + Gly + IPTG and M9 + Gly + Ara but could not on M9 + Gly + IPTG + Ara. These results indicated that the ectopic expression of VapBC15 with Lons or ClpXP2s caused growth arrest. Combined with the results of Western blot analyses for VapB15 proteolysis (Figure 5), we thus concluded that the ectopic production of Lons or ClpXP2s can activate the potential toxicity of the VapB15-VapC15 complex via degrading VapB15 and freeing up VapC15.

## 4. Discussion

*Synechocystis* PCC6803 is believed to have 69 chromosomal TA operons [26], yet they still remain largely uncharacterized. In the present study, the *vapBC15* operon is shown to encode a genuine TA system, VapBC15. In this TA system, VapC15 functions as a growth inhibitor and VapB15 as an antitoxin (Figure 1). Structure analysis by the 3DJIGSAW prediction tool [41] and the DALI server [42] shows that VapC15 is structurally homologous to several characterized VapC toxins (Figure S3A). For example, the toxin PAE2754 from *Pyrobaculum aerophilum* [43] has been identified to function as a sequence-specific ribonuclease [44]. Therefore, it is conceivable that VapC15, similar with its homologues, exerts the growth-arrest effect by its ribonuclease activity.

Although showing no conserved domain related to the known antitoxins, the VapB15 antitoxin can physically interacts with VapC15, forming the VapB15-VapC15 complex (Figure 2). Structure analysis has showed VapB15 with a distinct structural feature: a well-folded structure at the C-terminus of VapB15 and a disordered structure at its N-terminus (Figure S3B). Usually, the antitoxins of type II TA systems comprise a well-structured N-terminal domain and an unstructured C-terminal region, and the unstructured region mediates the binding of the antitoxin to its cognate toxin [14,15]. In the VapB15 antitoxin, the roles of the N- and C-terminal regions remains to be determined in the future. Our further analysis shows that only 34 homologs of VapB15 (sharing 33–58% amino acid identity) were found (Figure S3B), and distributed mainly in cyanobacteria and bacteroides (Table S2). Reverse search for conserved gene neighbors has revealed that the VapB15-like genes are adjacent to VapC-family toxin gene and also to the genes encoding other families of toxins, such as MazF and RelE (Table S2). These findings further support the notion that the different types of toxins are associated with various antitoxins in a mix and match principle [3,16].

Type II TA systems are auto-regulated at the transcription level by the TA components. Typically, antitoxins partially repress transcription of their TA operons by binding to one or more operator sites, and full repression is achieved only by the binding of TA complex [14,15]. Therefore, the toxin components can be considered as co-repressors of their own transcription because TA complexes bind to DNA more strongly than antitoxins alone [14]. The operator sites recognized by TA components generally comprises one or two palindrome sequences which overlaps the −10 and/or −35 promoter element recognized by RNA polymerase [25]. However, the VapB15 antitoxin shows to up-regulate the transcriptional activity of its own promoter *P_vapBC15_* by specifically binding to the promoter region, and the VapC15 toxin abolishes the auto-regulatory effect by destabilizing the binding (Figure 3 and Figure 4). Also, the *P_vapBC15_* promoter region has two pairs of palindrome sequences, PS1 and PS2 (Figure 4A). Nevertheless, the palindrome sequence PS1, but not PS2, is required for the binding of VapB15 to the promoter (Figure 4). Based on the data in this study, we guess that an unusual mechanism may be implicated in the regulation of the VapBC15 system. To elucidate the underlined mechanism, further investigations are needed for the transcriptional auto-regulation of the *vapBC15* operon and the response of the VapBC15 components to various environmental stresses.

Toxin activity and transcription of TA systems are both regulated by specific proteases which degrade antitoxin [14,45]. Proteolytic activation of TA systems has been studied in great detail in *E. coli* [45]. Among *E. coli* proteases, only Lon and ClpXP have been demonstrated to be engaged in antitoxin degradation. Most *E. coli* antitoxins are degraded by Lon or ClpXP, and only a few are known to be degraded by both [45]. Generally, specific stresses induce the proteases Lon and/or ClpXP, and then the degradation of antitoxin would cause the activation of TA systems. In *Synechocystis*, there exist only one Lon homolog (Lons) and eight Clp components, including four proteolytic subunits (ClpP1s, ClpP2s, ClpP3s, and ClpRs) and four ClpP ATPase-chaperones (ClpCs, ClpXs, ClpB1s, and ClpB2s) [46]. However, only ClpP2s and ClpXs, encoded by the dicistronic genes *sll0534* and *sll0535*, exhibit significant sequence identity (about 60%) with the ClpXP components of *E. coli*. Proteases involved in antitoxin degradation have generally been determined by comparing the level of antitoxin in protease-deficient strains to that in the wild-type strain using Western blot analysis. We have tried to construct the *Synechocystis* mutants deficient of Lons and respective Clp subunits but only succeeded in generating complete knockouts of *lons* and *clpB2s* (unpublished data). This is a reason that the selection-expression system of *E. coli* has been used to determine the role of Lons and ClpXP2s in antitoxin degradation. Using this strategy, it was demonstrated that ClpXP2s could specifically cleave the VapB10 antitoxin of VapBC10, but Lons could not [28]. Here, the artificial induction of ClpXP2s and Lons shows to degrade the free and the VapC15-bound VapB15, and thus activate VapC15 in the heterogenous host. Therefore, further studies are needed for the activation of proteases and the degradation of VapB15 in the native host under specific stress conditions.

In this study, we identified the VapBC15 TA system encoded by the *vapBC15* operon on the *Synechocystis* chromosome. In this system, VapB15, functioning as the antitoxin, displayed quite distinct characteristics from the described type II antitoxins, including the secondary structure, auto-regulatory activity, and proteolytic sensitivity. These unique properties of VapB15 constitutes the molecular basis of the regulation of VapBC15 activity. Therefore, this study increased our understanding of the regulatory mechanisms of type II TA systems and provided us with clues for further studying the function of VapBC15.

## Figures and Tables

**Table 1:** Strains and plasmids used in this study.

Strains/Plasmids	Genotype/Plasmid Characteristics *	Source or Reference
**Strains**		
*E. coli* DH5α	F^−^, φ80d *lacZ*ΔM15, Δ(*lacZYA*-*argF*) U169, *deoR*, *recA1*, *endA1*, *hsdR17*(r_k_^−^, m_k_^+^), *phoA*, *supE44*, λ^−^, *thi*-1, *gyrA96*, *relA1*	Stratagene (San Diego, CA, USA)
*E. coli* BL21(DE3)	F^−^, *ompT*, *hsdS*_B_(r_B_^−^ m_B_^−^), *gal*(λ *cI*857, *ind*1, *Sam*7, *nin*5, *lac*UV5-T7*gene*1) *dcm*(DE3), *lon*	Stratagene
*Synechocystis* sp. PCC 6803	wild type (WT)	ATCC 27184 (Manassas, VA, USA)
**Plasmids**		
pMD-18T	pUC replicon, Ap^r^	Takara Biotech (Dalian, China)
pET30a	pBR replicon, *lacI^q^*, *P_T7__lac_*, Km^r^	Novagen (Madison, WI, USA)
pJS298	pBR replicon, *P_T7lac_*, *P_BAD_*, Km^r^	[27]
pJS307	pJS298, *P_T7__lac_*-vapC15, Km^r^	This study
pJS357	pJS298, *P_T7__lac_*-vapC15, *P_BAD_*-*vapB15*, Km^r^	This study
pJS371	pJS298, *P_T7__lac_*-*lons*, Km^r^	[27]
pJS391	pJS298, *P_T7__lac_*-*clpxp2s*, Km^r^	[27]
pJS666	pET30a, *P_T7__lac_*-*vapBC15*, Km^r^	This study
pJS694	pMD-18T, *P_vapBC15_*, Ap^r^	This study
pJS744	pJS298, *P_BAD_*-*vapBC15*, *P_T7__lac_*-*lons*, Km^r^	This study
pJS745	pJS298, *P_BAD_*-*vapBC15*, *P_T7__lac_*-*clpxp2s*, Km^r^	This study
pJS759	p15 replicon, promoter-less *lacZ*, Sp^r^	[28]
pJS766	pMD-18T, *P_vapBC15_-vapB15*, Ap^r^	This study
pJS778	pJS759, *P_vapBC15_-lacZ*, Sp^r^	This study
pJS779	pJS759, *P_vapBC15_-vapB15-lacZ*, Sp^r^	This study
pJS882	pJS298, *P_BAD_*-*vapB15*, *P_T7__lac_*-*lons*, Km^r^	This study
pJS883	pJS298, *P_BAD_*-*vapB15*, *P_T7__lac_*-*clpxp2s*, Km^r^	This study
pJS956	pMD-18T, *P_vapBC15_-vapBC15*, Ap^r^	This study
pJS962	pJS759, *P_vapBC15_-vapBC15-lacZ*, Sp^r^	This study

* *lacZ*, β-galactosidase gene; Ap^r^, ampicillin resistance; Km^r^, kanamycin resistance; Sp^r^, spectinomycin resistance; pBR, pUC or p15, plasmid replicon.

## Supplementary Materials

The following are available online at http://www.mdpi.com/2073-4425/9/4/173/s1. Figure S1. Schematic diagram showing the genetic structure of the *vapBC15* operon. Figure S2. Matrix-assisted laser desorption/ionization-time-of-flight (MALDI-TOF) mass spectrometry analysis of VapB15 and VapC15-His_6_. Figure S3. Multiple structure-based sequence alignments of VapC15 and VapB15 homologues. Table S1. All primer sequences used in the present study. Table S2. A list of the blasted homologues of VapB15.
