# Supplementary material for: A Toxin-Antitoxin System VapBC15 from Synechocystis sp. PCC 6803 Shows Distinct Regulatory Features
Source: Genes (Basel). 2018 Mar 21;9(4):173. doi: 10.3390/genes9040173 (PMC5924515; doi:10.3390/genes9040173)
Supplement: Supplementary file 1 [file genes-09-00173-s001.zip › Supplementary files/Figure S1.docx]

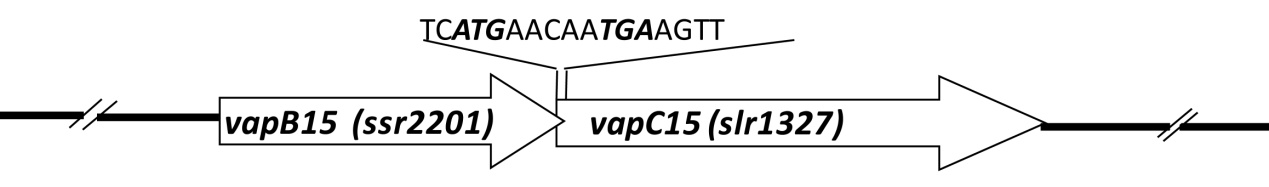


**Figure S1** Schematic diagram showing the genetic structure of the *vapBC15* operon. The 11 overlapping sequence of *vapB15* and *vapC15* are shown. The translation start coden ATG of *vapC15* and the end coden TGA of *vapB15* are in bold italic
