# Supplementary material for: A Toxin-Antitoxin System VapBC15 from Synechocystis sp. PCC 6803 Shows Distinct Regulatory Features
Source: Genes (Basel). 2018 Mar 21;9(4):173. doi: 10.3390/genes9040173 (PMC5924515; doi:10.3390/genes9040173)
Supplement: Supplementary file 1 [file genes-09-00173-s001.zip › Supplementary files/Figure S2.docx]

**
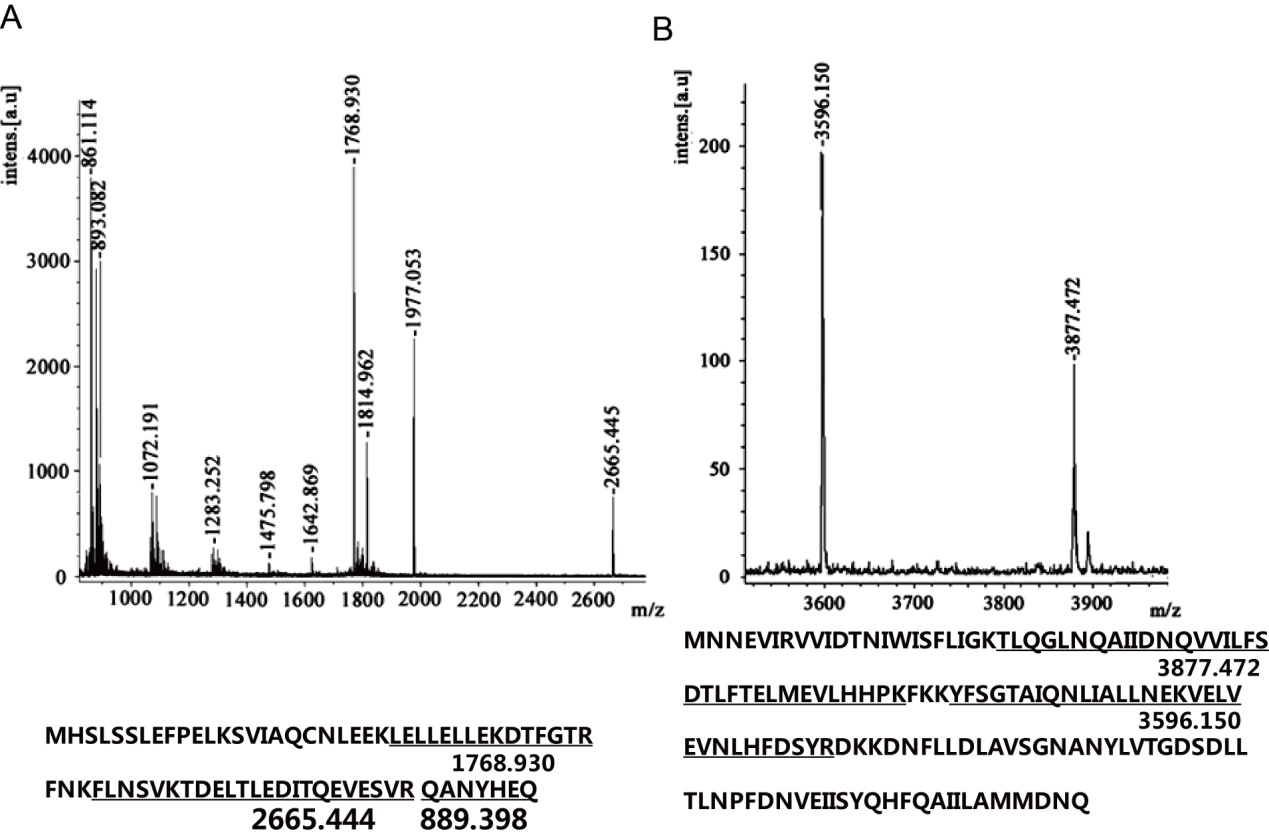
Figure S2** MALDI-TOF mass spectrometry analysis of VapB15 and VapC15-His_6_. A and B show the peptide mass fingerprintings of VapB15 and VapC15-His_6_ (Figure 2, lane 3), respectively. Amino acid sequence and predicted peptide fingerprint masses (m/z) by online analysis of VapB15 or VapC15-His_6_ are shown below.
