# Supplementary material for: A Toxin-Antitoxin System VapBC15 from Synechocystis sp. PCC 6803 Shows Distinct Regulatory Features
Source: Genes (Basel). 2018 Mar 21;9(4):173. doi: 10.3390/genes9040173 (PMC5924515; doi:10.3390/genes9040173)
Supplement: Supplementary file 1 [file genes-09-00173-s001.zip › Supplementary files/Figure S3.doc]

**Figure S3** Multiple structure-based sequence alignments of VapC15 and VapB15 homologues. Secondary structure is mapped onto the sequence, helices are indicated in red, strands are shown in green and the secondary structure elements represented above the alignment relate to the predicted structure of VapC15 (A) or VapB15 (B). Asterisks denote fully conserved residues in the alignment of the bacterial and archeal PIN domains. VapC_Mt5_ from *Mycobacterium tuberculosis*; VapC_Pyf_ from *Pyrococcus furiosus*; PAE2754 from *Pyrobaculum aerophilum*; VapC_Sfl_ from *Shigella flexneri*, FitB from *Neisseria gonorrhoeae*. WP_010872705(VapB15) from *Synechocystis sp*. PCC 6803; WP_011362783 from *Chlorobium chlorochromatii* CaD3; OQY57254 from *Desulfobacteraceae bacterium* 4572_88; WP_080808852 from *Candidatus Desulfamplus magnetomortis*; WP_009628504 from *Pseudanabaena biceps* PCC 7429; OIP76560 from *Oscillatoriales cyanobacterium* CG2_30_44_21; WP_013617930 from *Bacteroides salanitronis* DSM 18170; OFX58694 from *Bacteroidetes bacterium* GWA2_30_7; ETK03909 from *Tannerella sp*. oral taxon BU063 isolate Cell 6/7/9; WP_015178313 from *Oscillatoria nigroviridis* PCC 7112; WP_066346260 from *Geminocystis sp*. NIES-3708; WP_026794844 from *Planktothrix agardhii* NIVA-CYA 126/8; WP_072722569 from *Planktothrix tepida* PCC 9214; CUM59478 from *Planktothrix agardhii*; WP_054465013 from *Planktothricoides sp*. SR001; WP_008307984 from *Leptolyngbya sp*. PCC 6406; WP_073171954 from *Tangfeifania diversioriginum*; WP_008055499 from *Arthrospira sp*. PCC 8005; WP_006617886 from *Arthrospira platensis* NIES-39; WP_075890005 from *Limnothrix rosea* IAM M-220; WP_044522988 from *Nostoc sp*. PCC 7120; OKZ12094 from *Bacteroides oleiciplenus*; WP_022401255 from *Bacteroides uniformis* CAG:3; BAB74121 from *Nostoc sp*. PCC 7120; KXT51164 from *Bacteroides intestinalis*; WP_082788063 from *Bacteroides intestinalis*; WP_081795853 from *Bacteroides stercorirosoris*; WP_081743726 from *Bacteroides timonensis*; WP_080811692 from *Halomicronema hongdechloris* C2206; WP_009125549 from *Bacteroides fluxus* YIT 12057; WP_009132366 from *Bacteroides oleiciplenus* YIT 12058; WP_024283037 from *Algoriphagus marincola* HL-49; WP_007662927 from *Bacteroides intestinalis* CAG:564; WP_015215486 from *Anabaena cylindrica* PCC 7122; WP_009184126 from *Cecembia lonarensis* LW9.
