# Supplementary material for: A Toxin-Antitoxin System VapBC15 from Synechocystis sp. PCC 6803 Shows Distinct Regulatory Features
Source: Genes (Basel). 2018 Mar 21;9(4):173. doi: 10.3390/genes9040173 (PMC5924515; doi:10.3390/genes9040173)
Supplement: Supplementary file 1 [file genes-09-00173-s001.zip › Supplementary files/Table S1.doc]

**Table S1** All primer sequences used in present study

| Primers | Sequence (5′-3′) | Restriction site* |
| --- | --- | --- |
| ssr2201-N | GAGAACATATGCACTCATTGTCATCACTGG | *Nde* I |
| Ssr2201-S | GAGAAGAGCTCG CACTCATTGTCATCACTGG | Sac I |
| Ssr2201-K | GGAGAGGTACCGTGTAATACTTCCATCAGCTCAG | *Kpn* I |
| Slr1327-N | GAGGACATATGAACAATGAAGTTATCCGG | *Nde* I |
| slr1327-K | GAGAAGGTACCTACCCATGGTTTGGCGG | *Kpn* I |
| slr1327-X | GAGAACTCGAGCTGATTATCCATCATGGCTAAA | *Xho* I |
| ssr2201-1 | GGTGGACAGGGGGTTACTGG |  |
| ssr2201-2 | GAAATTCCAGTGATGACAATGAG |  |
| slr1327-1 | GGAGATCTAGAGTGACGGGAGATAGTGACC | *Xba* I |
| slr1327-2 | gagagaatTccGTGGAACTAGCCCAGGAC | *Eco*R I |
| P1-F | CAAAGCTCTGGCAACCCTGC |  |
| P2-F | GGGGACTTTTCAAGCAACTCC |  |
| P_BAD_-F | gtccacattgattatttgcacgg |  |
| P_BAD_-R | CTTCTCCTTTGCTAGCCATATG |  |

* The restriction sites added are underlined.
